# Disseminated learning from clinician-scientists: a multiple case study in physiotherapeutic care

**DOI:** 10.1186/s12909-018-1374-0

**Published:** 2018-11-23

**Authors:** Esther E. van Dijk, Manon Kluijtmans, Jonne P. Vulperhorst, Sanne F. Akkerman

**Affiliations:** 1 Education Center, University Medical Center Utrecht, HB4.05, PO Box 85500, Utrecht, The Netherlands; 2 Faculty of Social and Behavioral Sciences, Utrecht University, Utrecht, The Netherlands; 3 Centre for Academic Teaching, Utrecht University, Utrecht, The Netherlands

**Keywords:** Clinician-scientist, Boundary crossing, Disseminated learning, Physiotherapy, Multiple case study, Qualitative research

## Abstract

**Background:**

Clinician-Scientists are considered to be important for continuous improvement of patient care, because they are ideally positioned to bridge the gap between scientific research and clinical care. However, limited empirical evidence is available about how they connect these two realms. So far research has mainly focused on their direct role in bridging the gap. This study investigates an additional mechanism; that is whether clinician-scientists also connect science and care indirectly through disseminated learning. During this type of learning, clinical colleagues learn by working with clinician-scientists.

**Methods:**

Disseminated learning was studied in five physiotherapeutic care settings in the Netherlands with clinician-scientists (*N* = 5) and their clinical colleagues (*N* = 14). Semi-structured interviews were conducted between March and May of 2016. Interviews were transcribed verbatim and analyzed using thematic analysis.

**Results:**

Clinicians and clinician-scientists in all settings reported clinicians learning informally. They learned by being informed about (evidence for) new tests and treatments, through increased understanding of the research process and research results, and through awareness of an academic reflective approach to care. Learning took place primarily through knowledge sharing, and to a lesser extent through role modeling or joint implementation. Interpersonal and organizational conditions, such as overlapping clinical expertise and organizational policy and culture, seemed to facilitate or hinder learning.

**Conclusions:**

This study highlights disseminated learning as a mechanism of how clinician-scientists may connect science and care. Furthermore, it provides insight into how disseminated learning may take place and the conditions that may facilitate or restrict learning.

**Electronic supplementary material:**

The online version of this article (10.1186/s12909-018-1374-0) contains supplementary material, which is available to authorized users.

## Background

Concern about the poor connection between science and clinical care has been a longstanding issue [1]. For advancement of patient care and public health, it is crucial that clinically relevant questions are addressed in research and that results lead to the improvement of preventive and curative care. Clinician-Scientists, who combine research activities and clinical care, are important in connecting both realms. This connective role is widely recognized by the international health community, along with growing concern about the scarcity of clinician-scientists [2–5]. To improve education and professional performance of clinician-scientists, and to encourage students to choose this career path, it is important to gain better insight into the role of clinician-scientists as brokers between science and care.

Thus far, studies have focused on the direct role of clinician-scientists in connecting science and care, in the sense that clinician-scientists use their clinical experience to formulate relevant research questions and apply findings to improve their treatment of patients [6, 7]. However, clinician-scientists may also indirectly contribute to a connection between science and care, namely by inspiring their clinical colleagues who may learn from their particular perspective. We introduce the term disseminated learning to describe this process, which is in line with literature that argues professional learning often takes place in informal settings [8]. To our knowledge, no empirical studies have investigated the effect clinician-scientists may have on informal learning of clinicians.

### Boundary crossing theory and brokers

Boundary crossing theory can be used to explore the learning potential of clinician-scientists; this perspective describes them as brokers between two fields [9]. Brokers move between different contexts such as work and school, work and home life, or in the case of clinician-scientists, research and patient care. When moving between contexts, brokers may encounter discrepancies that lead to discontinuities in action or interaction (e.g., facing different professional standards may cause a professional to question what to do at work). Such discontinuities across contexts have been referred to as boundaries [10]. The position of brokers at these boundaries may be complicated, but also provides possibilities for connecting different contexts through learning. For clinician-scientists, this may happen when they generate a research proposal inspired by their clinical work or when they innovate clinical practice based on their research experiences. Literature on brokers suggests that they may also establish connections between contexts when they help people in one context learn something about another context that the people themselves do not participate in, for example, by providing resources and knowledge from that context [11, 12]. For clinician-scientists this implies that may exert their broker role not only directly by connecting science and practice in person, but also indirectly through connecting their clinical colleagues to the world of science through disseminated learning.

### Aim and research questions

Previous research from the perspective of boundary crossing has provided insight into how clinician-scientists combine and connect the professional practices of research and patient care [9]. This study intends to expand insights into clinician-scientists as brokers by exploring whether, and if so how, disseminated learning takes place in clinical settings where clinician-scientists are employed. We chose physiotherapeutic care as an example of such a setting. The primary research question was as follows: “Do clinicians experience disseminated learning?” Three sub-questions were formulated to explore in detail how disseminated learning might take place: “What do clinicians learn from their clinician-scientist colleagues?” “How do clinicians learn from their clinician-scientist colleagues?” and “What conditions facilitate or restrict learning?”

## Methods

### Design and procedure

We conducted a qualitative multiple case study [13] using semi-structured interviews. Interviews were conducted in March and May of 2016 by the first author. Each case consisted of one clinician-scientist and two to three of their clinical colleagues working within an organization providing physiotherapeutic care in the Netherlands. Clinician-Scientists were included because it was thought that from their outsider perspective, they might be able to identify subconscious clinician learning. Clinician-Scientists were recruited by sending an open mailing to second-year students and alumni of the Master of Science (MSc) program of Physiotherapy Sciences at Utrecht University to participate in the study. The e-mail explained the goals and design of the study, as well as the selection criterion for participation. Students and alumni were only eligible for participation if they at the time of the study or recently (< 6 months) combined clinical activities and research activities (e.g. PhD research or a substantial research project at minimally MSc level). All participating clinical colleagues were physiotherapists identified through nominations of clinician-scientist participants based on two criteria. Firstly, clinicians had to interact with their clinician-scientist colleagues at least twice a week. Secondly, clinicians could not have received extended formal research training, such as doctoral education or a health sciences MSc program, beyond the (limited) training that is an obligatory part of their (continued) clinical education. To prevent selection bias toward colleagues with more positive views, clinician-scientists were asked to nominate colleagues with expectedly different attitudes toward their scientific background; both skeptical and enthusiastic colleagues were included. Nominated clinical colleagues were approached by the researcher to participate in the study with an e-mail similar to the one used for clinician-scientists.

### Participants

Nineteen participants were interviewed individually in a private meeting room at their workplace. Interviews lasted between 23 and 42 min. Participants were five clinician-scientists (three men, two women) and 14 clinical colleagues (seven men, seven women) with an average age of 40 (range: 26–62 years). Clinician-Scientists were four alumni (two involved in PhD research, one in freelance research, and one recently finished her master’s thesis research) and one second-year student (involved in master’s thesis research) of the previously mentioned part-time MSc program in Physiotherapy Sciences. All participants, both clinician-scientists and clinicians, were licensed physiotherapists (national registration), which means they minimally hold a Bachelor of Science (BSc) degree in physiotherapy from a university of applied sciences in the Netherlands or an equivalent international degree. Organizations consisted of three primary care, one secondary care, and one tertiary care settings, all in different cities and provinces in the Netherlands. Two out of five organizations employed other clinician-scientists along with the clinician-scientist participating in the study. A description of each organization is shown in Table 1.

**Table 1:** Description of type and composition of the organizations used as cases

Organization	Type of organization (I, primary care; II, secondary care; III, tertiary care setting)	Participating clinician-scientists	Participating clinicians	Presence of other clinician-scientists within the organization
A	Specialized physiotherapy practice (II)	1	3	No
B	Physiotherapy practice (I)	1	2	Yes
C	Physiotherapy practice (I)	1	3	No
D	Physiotherapy practice (I)	1	3	No
E	Department in a university medical center (III)	1	3	Yes

### Materials

Separate interview guides, but with similar themes were employed for interviews with clinician-scientist and clinician participants (the interview guide can be found in Additional file 1). Interview guides were structured around our three research sub-questions and based on literature about learning outcomes [14], learning processes [15] and organizational circumstances that influence learning [11]. Interview guides can be found in the supplementary materials.

### Data analysis

Interviews were audio recorded and transcribed verbatim. The data were analyzed using thematic analysis [16]. Data analysis began with familiarization and producing initial in-vivo codes and was then followed by a cyclic process of data checking and reviewing codes. Coding was done by ED, with regular code checks by MK, using the analytical software package “Dedoose” (version 7.6.6, published by SocioCultural Research Consultants, CA, USA). Through a recursive process of peer debriefing [17] and regular discussions amongst all authors, final themes were determined. These themes were subsequently studied separately for clinician and clinician-scientist perspectives. The final code tree corresponds to the leveled headings in the results section and is available upon request. Finally, we conducted a co-occurrence analysis in Dedoose, which provides an overview of excerpts with co-occurring codes [18, 19], to examine possible relationships between what and how clinicians learn. By specifically analyzing the excerpts with co-occurring codes from both themes we could extend our analysis by exploring connections between what and how clinicians learn.

### Ethical considerations

This study was approved February 2016 by the Ethical Review Board of the Netherlands Association for Medical Education (Nederlandse Vereniging voor Medische Onderwijs [NVMO]) (identification number 654). Written informed consent was obtained from all participants. To ensure anonymity but enhance readability, occupation and numbers are used in quotes throughout this paper.

## Results

In all five organizations, both clinicians and clinician-scientists reported that clinicians learned informally from their clinician-scientist colleagues. For each of the sub-questions, we report the findings per theme, first providing the clinician perspective and then the clinician-scientist perspective.

### What do clinicians learn from their clinician-scientist colleagues?

Three themes emerged in the data regarding what clinicians learn from their clinician-scientist colleagues: science as resource, science as process, and science as perspective.

#### Science as resource

The clinicians learned from their clinician-scientist colleagues through descriptions and explanations of scientific evidence and through access to scientific literature, thus improving clinicians’ access to science as a resource for informing and innovating clinical practice.

Clinicians also reported learning from their clinician-scientist colleagues about new tests and treatments or improvements to existing tests and treatments. Some clinicians reported not only learning how to apply a test or treatment, but also why a particular test or treatment was preferred.

When I asked “Why would you do this rather than that?” he answered, for example, “Research has shown that treatment A is effective and treatment B is not.” – Clinician 2, Organization A

Clinicians described that clinician-scientists played an important role in changing the way they diagnosed and treated patients by altering care protocols. In addition, clinician-scientists provided information about why a protocol was changed, especially with regard to evidence supporting or discouraging the use of specific tests and treatments in old and new protocols.

Lastly, within the science as resource theme, clinicians reported learning when their clinician-scientist colleague suggested and forwarded relevant scientific information sources, such as research articles or books.

We have a project here called [name of the project]. She told us about research from TNO [on this subject]. And besides that, we often get information from her about research that has been conducted. – Clinician 6, Organization C

The clinician-scientists described this domain of learning by their colleagues as resulting from active effort on their part. They not only deliberately shared their knowledge of research results and forwarded scientific information sources to their clinical colleagues, but also purposely filtered their information (sources) for relevance to their clinical colleagues.

[I share information with clinical colleagues] when I read an article which I believe to be of particular interest or benefit to someone else’s work and when I’m discussing a patient [with a colleague] and I suddenly remember something I have read or learned. – Clinician-Scientist 5, Organization E

#### Science as process

The clinicians described gaining insight into the scientific research process via their clinician-scientist colleagues. This theme concerns knowledge and understanding, as well as an altered attitude toward science. Clinicians reported that their clinician-scientist colleagues helped them to understand research papers by explaining research methods, research design, the structure of a research paper, or specific terms used in research papers.

[My clinician-scientist colleague] will be more likely to look at the design, variation and substantiation [of research methods] and she shares that knowledge with us, so I think that’s what I learn from her. – Clinician 14, Organization E

Less commonly, clinicians reported developing a different attitude toward science because of their clinician-scientist colleagues. Some described an increased appreciation for research; however, most describe no changes. One clinician that did describe a different attitude expressed this change as follows:

Perhaps he’s made me somewhat more conscious of the usefulness of research. – Clinician 2, Organization A

Clinician-Scientist perceptions aligned with the perspectives of their clinical colleagues: they described helping their colleagues to understand research papers and research methods. Additionally, they indicated that their clinical colleagues gained a better understanding of the amount of work that goes into research because of them. For example, one clinician-scientist explained that collecting data for his research made his colleagues realize the following:

That it [scientific research] takes time, that you really should put in the work. And that it also demands effort from people working in the clinic. – Clinician-Scientist 1, Organization A

In contrast to the reporting of some of the clinical colleagues, the clinician-scientists themselves did not expect or notice they had an impact on clinicians’ attitudes toward science in the sense of increasing their clinical colleagues’ appreciation for science.

#### Science as perspective

Third, the clinicians reported learning how to reflect on clinical practice from the academic perspective of their clinician-scientist colleagues. Some clinicians explicitly reported that their clinician-scientist colleagues had stimulated them to take on an academic perspective themselves.

Why do you do this? And why that? The why-question. And the reasoning behind what you’re doing. That arises more often. I believe I am motivated [in that area] by [clinician-scientist] colleagues. – Clinician 4, Organization B

Clinicians from one organization described the ability of their clinician-scientist colleagues to reflect on their work from a high abstraction level and put the organization’s clinical practice into an overall perspective.

When we are talking in our team, [Clinician-Scientist 3] regularly is the one, which can be noticed from her remarks, who looks at the bigger picture. – Clinician 6, Organization C

This domain of learning was also reported by the clinician-scientists. They described promoting the importance of looking from a critical and overall perspective and substantiating clinical reasoning and decisions. They also described how they went about this. Clinician-Scientists explicitly expressed the hope that their clinical colleagues would also do this themselves.

### How do clinicians learn from their clinician-scientist colleagues?

We found evidence for three learning processes: sharing, role modeling, and joint implementation. A co-occurrence analysis was used to relate learning types to learning processes (Table 2). The frequencies of the co-occurring codes indicate that learning happens mostly through sharing, and to a lesser extent through role modeling and joint implementation.

**Table 2:** Co-occurrence analysis for learning domains and learning processes

	Sharing	Role modeling	Joint implementation
Science as resource	21	8	4
Evidence for tests and treatments	9	2	4
New or adjusted tests and treatments	8	6	0
Access to relevant information	2	0	0
Altering guidelines or protocols	2	0	0
Science as process	9	0	0
Understanding research methods	2	0	0
Understanding scientific papers	5	0	0
Appreciating the value of research	1	0	0
Understanding the research process	1	0	0
Science as perspective	2	11	0
Overall perspective	0	5	0
Substantiating (clinical) reasoning and choices	2	6	0

#### Sharing

The clinicians described learning from clinician-scientist colleagues when their clinician-scientist colleagues shared information with them. The co-occurrence analysis indicated that sharing was an important learning process for learning within the themes of science as resource and science as process. Both the clinicians and clinician-scientists could initiate sharing. Clinicians indicated that clinician-scientists shared their knowledge with them through informal conversations and e-mails, as well as during formal team meetings, such as journal clubs or case discussions.

If I’m stuck on something or I’m having trouble moving forward with a particular patient, then I might walk over to him and say: “This is what I’ve done. Do you have any tips, tricks?” And then he says to me: “I’ve read something about that, maybe you can try it.” – Clinician 10, Organization D

The descriptions of sharing from the clinician-scientists corresponded to those of their clinical colleagues. Clinician-Scientists provided further explanations about how they shared information: they did so through forwarding information sources, translating scientific information, or adapting protocols. The following quote demonstrates the translation:

It’s not relevant for my colleagues to know what statistical test or method is used to draw a conclusion. But in the end, the conclusion is very interesting, as well as why something is the better option. – Clinician-Scientist 1, Organization A

#### Role modeling

The clinicians also learned through role modeling, or observation and imitation of clinician-scientist colleagues. The co-occurrence analysis suggested that this learning process was related to learning about (evidence for) new tests and treatments and learning within the theme of science as perspective. Clinicians described learning about new tests and treatments from their clinician-scientist colleagues when they saw them using them in practice, and asked about them.

I see [my clinician-scientist colleague] carrying out a particular exercise and I think: OK, he does it this way. Then I often ask for background information, for example: “Hey, what are you doing with the patient exactly? And why did you choose that particular exercise?” – Clinician 11, Organization D

Role modeling is also related to developing an academic perspective toward clinical practice. Clinicians seemed to learn from observing how their clinician-scientist colleagues reflected on clinical practice, such as how they substantiated their clinical performance or how they kept in mind meta-level considerations:

I think it’s interesting to observe how she can see the bigger picture. How she does it. And hence I learn (…) how to look further than my daily [clinical] practice. – Clinician 6, Organization C

Role modeling as a learning process and its relation to the same learning types were also indicated by the clinician-scientists. They further described explicating their scientific perspective to their clinical colleagues and encouraging them to use this perspective themselves.

[Colleagues can learn from me] to view things from another perspective. That I say [to them]: “Hey, you can also look at it in this way.” And my colleagues then say: “Oh, right, that’s also possible, to look at it more like that.” – Clinician-Scientist 3, Organization C

#### Joint implementation

Least frequently, clinicians described learning about (evidence for) tests and treatments from clinician-scientists through jointly implementing a new way of treating or testing within their organization.

She comes across subjects and thinks: “Hey, that’s important.” And then we try to implement that together. – Clinician 7, Organization C

Joint implementation of projects was also reported by clinician-scientists.

### What conditions facilitate or restrict learning?

Conditions that seemed to facilitate and restrict learning could be divided in two categories: interpersonal and organizational.

#### Interpersonal level

Two interpersonal conditions were reported, both by clinicians and clinician-scientists. *Overlapping clinical expertise*, (e.g., specialization in “psychosomatic conditions” or “sports injuries”), facilitated learning, because there was more frequent contact and because knowledge of clinician-scientists was more relevant. A quote from a clinician-scientist illustrates this:

I can learn plenty from her about neurology, but I don’t see patients with neurological conditions. […] So, with regard to content knowledge, we don’t learn a lot from each other, because we both have our own specialization. – Clinician-Scientist 2, Organization C

*Clinician awareness of clinician-scientists experience in science* was also reported to increase learning opportunities, as clinicians were more inclined to ask for their additional, scientific, perspective.

#### Organizational level

Organizations that were described by participants as having an *organizational policy and culture* that expressed an appreciation for science were reported to facilitate learning. These organizations explicitly created learning opportunities, both by actively organizing activities such as journal clubs and by implicitly expecting clinicians to be interested in evidence-based practice and science. It was also suggested that organizations with an expressed appreciation for science were likely to attract and employ more clinician-scientists and more clinicians with an interest in science. *Formalized broker roles,* in which clinician-scientists spent less time on patient care and were provided with time to use their science background to innovate clinical practice, were described as a way for clinician-scientists to stimulate clinician learning, for example through implementation of new tests and treatment methods. *Time constraints*, often imposed by high patient loads, were reported to hinder learning from clinician-scientists, as they limited the available time for knowledge sharing.

That you have insufficient time to share that [knowledge]. That is in fact a great pity […] You could learn much more from each other, considerably more. – Clinician 12, Organization E

Additionally, clinician-scientists reported that *systematically storing sources concerning evidence to support evidence-based practice*, for example scientific articles or (updates for) evidence-based treatment guidelines, may potentially facilitate learning. This enables clinicians to receive and retrieve relevant scientific information that clinician-scientists have supplied to their organization.

## Discussion

To our knowledge, this study is the first to empirically investigate the assumption of clinician-scientists bridging role in informal learning of clinical colleagues. Our results show that, at least in the field of physiotherapy in the Netherlands, clinicians learn informally from their clinician-scientist colleagues, as learning was reported across a variety of physiotherapeutic care settings by both clinicians and clinician-scientists. This sheds new light on clinician-scientists’ valuable role in connecting the worlds of science and care, which is thought to be of importance to improve patient care. This study also contributes to boundary crossing literature by empirically evaluating the role of brokers from the perspective of people within one of the contexts the broker moves between. Although learning from brokers has already been suggested by others [11], our study provides the first evidence that people within one context learning from brokers is a mechanism for brokers to connect different contexts.

To gain more detailed insight into disseminated learning, we first explored what clinicians learn from their clinician-scientist colleagues. We found three learning domains: science as resource, science as process, and science as perspective. These domains differ in the extent to which they directly translate into patient care. When clinicians learn about (evidence for) tests and treatments from their clinician-scientist colleagues, this has a direct impact on patient care, because performing tests and treatments is an important part of their clinical work. Learning about the research process and applying an academic perspective on clinical practice areas is indirectly related to the care of patients, as it requires more active involvement on the part of clinicians to translate this learning into improvement of their everyday clinical practice. Adopting the academic perspective of clinician-scientists may help to stimulate critical thinking skills of clinicians, which is thought to improve the quality of their judgments and decisions [20]. Therefore, a more reflective and critical attitude could be expected to contribute to improved quality and innovation. Although the primary focus of this study was the clinical setting of clinician-scientists, it is important to note that improved understanding and attitudes of clinical colleagues toward research may also benefit research, for example through increased willingness to participate and quality of data captured for research in clinical settings.

Our second sub-question asked how clinicians learn from clinician-scientist colleagues. We identified three mechanisms for disseminated learning: sharing, role modeling, and joint implementation. Of these, sharing seemed the most important learning mechanism for all three learning domains. Literature on workplace learning suggests that informal learning often takes place without conscious effort and mostly concerns tacit knowledge [8, 21]. In addition to that literature, clinician-scientists in this study reported targeted efforts, such as making their information, knowledge, and experiences more comprehensible to clinical colleagues. This confirms the ideas of Meyer [11], who argues that brokers are able to translate knowledge to make it more accessible.

In our third sub-question, we explored organizational conditions that enhance or hamper disseminated learning. We found that disseminated learning may be enhanced by overlapping clinical expertise, clinician awareness of clinician-scientist expertise, a science-appreciative organizational policy and culture, formalized clinician-scientist roles, and systematic information storage, whereas time constraints were perceived as hindering. As we only studied a limited number of organizations, these results should be considered preliminary and further research into conditions for disseminated learning is needed. The identified conditions nevertheless show parallels with existing literature regarding conditions for workplace learning: awareness of clinician-scientist expertise relates to awareness of knowledge and expertise [22], organizational culture and policy demonstrating support for science can be linked to support for learning [23], time constraints correspond to lack of time [24] and systematically storing sources concerning evidence-based practice and science is connected to information systems [25].

This study comes with some limitations. First, our study was confined to cases from physiotherapeutic practice in the Netherlands. This means that one should be cautious to generalize our results to other countries as well as to clinicians and clinician-scientists from other disciplines. The amount of scientific training during clinical education may vary between programs, for instance physician training in the Netherlands is offered at research universities whereas physiotherapy training is offered at universities of applied sciences. However, in general clinician-scientists will have more extensive research training and experience, regardless of discipline or country. Therefore, a difference in scientific competencies may be expected, which provides potential for disseminated learning as found in our study. To confirm our results, we propose further research in a broader group of clinician-scientists and their respective clinical colleagues in a variety of countries, health care contexts and disciplines. As a next step, we recommend to study physician-scientists, as they constitute the largest group of clinician-scientists [4]. In addition, it is of interest whether disseminated learning from clinician-scientists extends to non-corresponding clinical professions, as this may be of major importance in inter-professional clinical care (e.g. nurses and physiotherapists learning about science from physician-scientists).

A second limitation is that we only focused on self-reported learning, and did not observe or measure actual changes in behavior, clinical practice, or patient outcomes. Measuring these types of change requires sophisticated research designs, such as observational or experimental studies, and would be an important next step in establishing the impact of disseminated learning.

Lastly, our sampling method may have caused a possible voluntary response bias. We sent an open invitation to the community of all alumni and second year students of the Physiotherapy Sciences program to increase our chances of recruiting enough participants, because clinician-scientists in general and non-physician clinician-scientists in particular are scarce [4]. Even though we could recruit enough participants using this method, it also increased our chances of an overrepresentation of clinician-scientists that are positive towards their influence on clinical colleagues in our sample.

Notwithstanding these limitations, this study confirms the importance of clinician-scientists in bringing scientific knowledge and perspectives into clinical care. This study also provides suggestions for organizations on how they can stimulate disseminated learning, which includes recognizing and formalizing the broker role of clinician-scientists. Active effort on the part of clinician-scientists in facilitating collegial learning also implies that investing in their roles as brokers, as well as communicative and/or didactic training, could be part of clinician-scientist training program, as it could increase their effectiveness as brokers in contributing to quality of care.

## Conclusions

This multiple case study in physiotherapeutic care settings in the Netherlands shows that informal learning of clinicians from clinician-scientists takes place and concerns both increased knowledge and application of evidence for tests and treatments (science as resource), improved understanding and appreciation of the research process (science as process), and awareness and sometimes application of a reflective academic approach to care (science as perspective). Learning happens mainly through information sharing and role modeling and less frequently through joint implementation. We conclude that disseminated learning seems to be one of the mechanisms through which clinician-scientists contribute to connecting science and care.

## Additional file

Additional file 1: Interview Guides (1 and 2). (DOCX 27 kb)

## Abbreviations

BSc: Bachelor of Science
COREQ: Consolidated criteria for reporting qualitative research
MSc: Master of Science
NVMO: Netherlands Association for Medical Education (Nederlandse Vereniging voor Medische Onderwijs)
TNO: Netherlands Organisation for Applied Scientific Research (Nederlandse Organisatie voor toegepast-natuurwetenschappelijk onderzoek)
