# Supplementary material for: Disseminated learning from clinician-scientists: a multiple case study in physiotherapeutic care
Source: BMC Med Educ. 2018 Nov 23;18:279. doi: 10.1186/s12909-018-1374-0 (PMC6260853; doi:10.1186/s12909-018-1374-0)
Supplement: Supplementary file 1 — Interview Guides (1 and 2). (DOCX 27 kb) [file 12909_2018_1374_MOESM1_ESM.docx]

**Additional file 1**

**INTERVIEW GUIDE 1: CLINICIANS**

**Introduction**

- Introduce yourself as researcher
- Describe the goal of the study
- Explain the structure of the interview
- Ask if there are any questions
- Obtain written informed consent

**Topic 1: Professional background and professional relationship with clinician-scientist colleague**

*My first topic concerns your professional background and your professional relationship with your clinician-scientist colleague.*

- What responsibilities do you have as a physiotherapist at your organization?
- Wat did your higher education consist of?
- Are you currently undertaking research training or a research project?
- How long have you and your colleague been working together?
- How often do you interact with him/her per week? When do you see him/her?
- Are you aware that your colleague studies/studied Physiotherapy Sciences and has conducted/is conducting scientific research?
  - Has this impacted you in any way?
  - How has this impacted you?

**Topic 2: Contact with clinician-scientist colleague *(learning process)***

*I would also like to know if you and your colleague ever discuss topics related to physiotherapy science, for example his/her research, scientific articles or your opinions about evidence-based practice.*

- Do you have conversations about these types of topics with your clinician-scientist colleague?
  - What are these conversations like? When do they take place?
  - To what extent does your clinician-scientist colleague share his/her experiences and ideas regarding physiotherapy science with you? To what extent is this new information to you? (*sharing)*
  - To what extent are these conversations characterized by contradictions and disagreement? *(constructive conflicts)*
  - To what extent are these conversations characterized by joint exploration of the topic? (*co-construction)*

**Topic 3: Clinician-scientists’ influence on clinical colleagues (*learning content/learning outcomes*)**

*With my next set of questions I aim to understand if and how the previously discussed contact with your clinician-scientist colleague impacts you professionally.*

- Do you feel you have picked up or adopted something from his/her scientific background?
  - What have you picked up or adopted from your colleague?
  - Do you know how and why this happened?
  - Has this impacted you professionally and if so, in what way?
- How does your organization decide on their working methods, for example with regards to treatment methods, tasks or administration of patient information? (*skills*)
  - What role do you have in this decision-making process? And what is your clinician-scientist colleagues role?
  - Has your clinician-scientist colleague caused you/your colleagues to do things differently?
  - If so, to what extent did his/her scientific background have anything to do with it?
- How is knowledge kept up to date within your organization and how is new knowledge acquired? (*knowledge*)
  - What role do you have therein? And your clinician-scientist colleague?
  - Have you ever acquired new knowledge from your clinician-scientist colleague? What did you learn?
  - If so, to what extent did his/her scientific background have anything to do with it?
  - In what type of situations did you acquire new knowledge from your clinician-scientist colleague? What did you learn?
  - Has this impacted you professionally and if so, in what way?
- What is your attitude towards physiotherapy science and physiotherapy-scientists? (*attitudes*)
  - Has your attitude towards physiotherapy science and physiotherapy-scientists changed because of your clinician-scientist colleague?
  - What has changed?
  - How has your clinician-scientist colleague changed your attitude?
  - Has this impacted you professionally and if so, in what way?

**Conclude the interview**

- Summarize the main points of the interview and check with participant
- Ask whether the participant has any comments or questions about the interview
- Ask whether the participant has any remaining questions about the study
- Thank participant for participating in the study

**INTERVIEW GUIDE 2: CLINICIAN-SCIENTISTS**

**Introduction**

- Introduce yourself as researcher
- Describe the goal of the study
- Explain the structure of the interview
- Ask if there are any questions
- Obtain written informed consent

**Topic 1: Professional background and professional relationship with clinical colleagues**

*My first topic concerns your professional background and your professional relationship with your clinician colleagues.*

- What are your responsibilities at the organization for which you work as a clinician/researcher? How many days do you work for your clinical/research organizations? How long have you worked at your clinical/research organization?
- Why did you decide to study Physiotherapy Sciences? Did you work at your current organization before/during your enrolment in the Physiotherapy Science program?
- Are your colleagues aware that you have studied/are studying Physiotherapy Sciences and have conducted/are conducting scientific research?
  - Does this impact your colleagues?
  - How does this impact your colleagues?

**Topic 2: Contact with clinical colleagues**

*I would also like to know if you and your colleagues ever discuss topics related to physiotherapy science, for example your research, scientific articles or your opinions about evidence-based practice.*

- Do you have conversations about these types of topics with your clinical colleagues? If so:
  - What are these conversations like? When do they take place?
  - To what extent do you share your experiences and ideas regarding physiotherapy science with your clinical colleagues? (*sharing*)
  - To what extent are these conversations characterized by contradictions and disagreement? *(constructive conflicts)*
  - To what extent are these conversations characterized by joint exploration of the topic? (*co-construction)*

**Topic 3: Clinician-scientists’ influence on clinical colleagues (*learning content/learning outcomes*)** *With my next set of questions I aim to understand if and how the previously discussed contact with your clinical colleagues impacts them professionally.*

- Do you feel your colleagues pick up or adopt certain things from you because of your scientific background and research activities ?
- If so:
  - What do colleagues pick up or have adopted from you?
  - Do you know how and why this happened?
  - Has this impacted them professionally, if so, in what way?
- How does your organization decide on their working methods, for example with regards to treatment methods, tasks or administration of patient information? (*skills*)
  - What role do you have in this?
  - Have your colleagues ever changed the way they work because of you?
  - If so, to what extent did your scientific background have anything to do with this?
- How is knowledge kept up to date within your organization and how is new knowledge acquired? (*knowledge*)
  - Have your colleagues acquired new knowledge because of you? If so:
  - What did they learn?
  - To what extent did your scientific background have anything to do with it?
  - In what type of situations have your colleagues acquired new knowledge because of you?
  - Does the new knowledge impact your colleagues professionally and if so, in what way?
- How would you describe your clinical colleagues attitudes towards physiotherapy science and physiotherapy-scientists? (*attitudes*)
  - Have your clinical colleagues attitudes towards physiotherapy science and physiotherapy-scientists changed because of you? If so:
  - What has changed?
  - How have you changed their attitude?
  - Has this impacted them professionally and if so, in what way?

**Topic 4: Factors influencing clinician learning**

- Are there factors that enhance or hinder clinical colleagues to pick up or adopt something from your scientific training and experience?
- If so:
  - What are these factors?
  - How do these factors enhance or hinder your colleagues to learn from your scientific training and experience?
- At the start of the interview you indicated that [paraphrase to what extent colleagues are aware of the clinician-scientists experience and training in research] *(awareness scientific background)*
  - To what extent has this awareness impacted whether your clinical colleagues pick up things from you? If so, can you describe this impact?
- How is (new) scientific information and knowledge stored within your organization? (*knowledge management system).* Is it easy to retrieve this information for you and your clinical colleagues?
- To what extent does this impact whether your clinical colleagues pick up things from you? If so, can you describe this impact?

**Conclude the interview**

- Summarize the main points of the interview and check with participant
- Ask whether the participant has any comments or questions about the interview
- Ask whether the participant has any remaining questions about the study
- Thank participant for participating in the study
